# Breeding in Its Relations to Physiology and Pathology

**Published:** 1881-07

**Authors:** 


BREEDING IN ITS RELATIONS TO PHYSIOLOGY AND PATHOLOGY. —The exact connection between comparative medicine and the very healthy Jersey cow, pictured on page 198, may not be at first apparent. But we are of the opinion that breeding, when scientifically investigated, will reveal much to the physiologist and pathologist. Breeding has already been well studied empirically. The art is well developed and perfected; the science, too, has been studied in certain limited directions by naturalists. But on the whole the correlative, physiological and pathological processes are very little understood. We hope to obtain a series of articles bearing on this aspect of the subject; especially with reference to its pathology and the physiological limits to which breeding can be carried.

The Jersey cow illustrates, as well as any animal, what breeding can do to develop a special function. The difference between the ordinary cow and a typical Jersey, as regards the size and activity of the mammary gland, is immense. Some of the best cows look actually deformed. Yet the limit of this glandular development does not seem yet to be reached. At the present rate it seems not unlikely that in time the udder will carry the cow instead of the cow the udder!

